# Field Distortion and Optimization of a Vapor Cell in Rydberg Atom-Based Radio-Frequency Electric Field Measurement

**DOI:** 10.3390/s18103205

**Published:** 2018-09-22

**Authors:** Zhenfei Song, Wanfeng Zhang, Qi Wu, Huihui Mu, Xiaochi Liu, Linjie Zhang, Jifeng Qu

**Affiliations:** 1 National Institute of Metrology, Beijing 100029, China; zhangwf@nim.ac.cn (W.Z.); skzxmhh@163.com (H.M.); liuxch@nim.ac.cn (X.L.); qujf@nim.ac.cn (J.Q.); 2 School of Electronic and Information Engineering, Beihang University, Beijing 100083, China; 3 School of Electronic Science and Engineering, Southeast University, Nanjing 210009, China; 4 Institute of Laser Spectroscopy, Shanxi University, Taiyuan 030006, China; zlj@sxu.edu.cn

**Keywords:** Rydberg atoms, electromagnetically-induced transparency (EIT), radio-frequency (RF) electric field, vapor cell, measurement uncertainty

## Abstract

Highly excited Rydberg atoms in a room-temperature vapor cell are promising for developing a radio-frequency (RF) electric field (E-field) sensor and relevant measurement standards with high accuracy and sensitivity. The all-optical sensing approach is based on electromagnetically-induced transparency and Autler-Townes splitting induced by the RF E-field. Systematic investigation of measurement uncertainty is of great importance for developing a national measurement standard. The presence of a dielectric vapor cell containing alkali atoms changes the magnitude, polarization, and spatial distribution of the incident RF field. In this paper, the field distortion of rubidium vapor cells is investigated, in terms of both field strength distortion and depolarization. Full-wave numerical simulation and analysis are employed to determine general optimization solutions for minimizing such distortion and validated by measuring the E-field vector distribution inside different vapor cells. This work can improve the accuracy of atom-based RF E-field measurements and contributes to the development of related RF quantum sensors.

## 1. Introduction

Atom-based quantum effects have been widely used in developing quantum sensors and metrology standards for various physical quantities, ranging from time and frequency, to magnetic and electric fields (E-fields), to voltage and current, to temperature and pressure [1,2]. Atomic sensors possess advantages including low field intrusion, good reproducibility, high accuracy and sensitivity, direct traceability to the International System of Units (SI), and atom-based self-calibration. Thus, they are promising for developing metrology standards as well.

Rydberg atoms are a class of atoms in highly excited states with one or more electrons of large principal quantum numbers [3]. Such atoms have peculiar properties, including high polarizability, high transition dipole moment, and sensitive responses to external electromagnetic fields, ranging from several to hundreds of gigahertz (GHz). Rydberg atoms are now intensively studied in quantum information and precision measurement [4]. The electromagnetically-induced transparency (EIT) [5,6] of a probe laser is prepared in a three-level Rydberg quantum system, and the applied radio-frequency (RF) E-field causes a strong Rabi oscillation between high-lying Rydberg states, which allows a new method of detecting field strength by measuring the ac-Stark induced Autler-Townes splitting [7] (an optical frequency quantity) within the probe transparency window. Independent of conventional dipole-based E-field sensing, the new technique has many advantages, as summarized in Table 1. The quantum coherence effect induced by a RF E-field in Rydberg atoms provides a promising solution for developing quantum E-field sensors and metrology standards [8,9,10].

Measurement error investigation and uncertainty evaluation are key aspects in developing a metrology standard. Sedlacek et al. first proposed a list of measurement error sources [8], and a quantitative description of systematic deviations from the linear relationship has been discussed by Holloway [20]. The quantitative investigation of other error sources is still in demand.

In this paper, we discuss the RF field distortion effect of a vapor cell, which is usually used for containing alkali atoms. The vapor cell is normally made of some non-metallic material, such as glass or quartz, and some micro vapor cells can be fabricated, so the field perturbation of a vapor cell is relatively small compared with antennas or commercial RF field probes. However, for precision measurement, the RF cavity resonance and electromagnetic scattering of the vapor cell still causes field distribution inside the cell that is different from the external incident field, and both the field strength and the polarization are changed. These alterations mean that the actual E-field interacting with the Rydberg atoms in the vapor cell is different from the incident field due to the RF cavity resonance and scattering. As a result, the measurement accuracy and effectiveness of traceability are largely affected by the vapor cells.

Regarding the field strength distortion, the effect of vapor cell geometry has been investigated by Fan [21]. It can be reduced by diminishing the *D*/*λ* ratio, where *D* is the inner size of the vapor cell, and *λ* is the wavelength of the RF field. A rule of thumb is that, for a cuboidal cell, the field variation is approximately 1% as long as the *D*/*λ* ratio is smaller than 0.1. Based on this conclusion, it remains hard to find an extremely small vapor cell for millimeter-wave or THz field sensing, and using a small vapor cell would also reduce the number of atoms and hence limit the measurement sensitivity. Alternatively, sometimes we can correct such field distortion by numerical simulation, or optimizing a point within a vapor cell volume for laser illumination [22]. However, this still results in large uncertainty, and the traceability of a numerical simulation is unclear. In this work, the effect of the vapor cell is further studied, both numerically and experimentally. We determine that cavity eigenmode frequency can be used as an important indicator of field distortion, and for the same *D*/*λ* ratio, the field variation inside the vapor cell depends on the wall thickness of the vapor cell. The lower eigenmode frequencies can be increased not only by reducing the *D*/*λ* ratio but also by reducing the wall thickness. This is validated by measuring the field distribution inside different vapor cells.

The E-field depolarization effect of a vapor cell is investigated as well. This starts with a study of the dependence of the probe transmission spectroscopy on the relative angle of the laser polarization and E-field vector, and the potential impact on E-field strength determination is revealed by a measurement at 15.09 GHz. We also show that the cavity resonance and E-field depolarization are not evenly distributed, but directly related to the position inside a vapor cell. This is validated by measuring the E-field vector distribution when scanning the lasers, and by finite-element electromagnetic modelling as well. Through experiments, a preliminary conclusion is reached that the depolarization effect in the middle of a vapor cell following the E-field polarization direction is less than that in other positions. This research provides guidance for promoting the atom-based RF E-field measurement, which may be useful for other related quantum sensing in RF fields.

## 2. Theory and Methods

### 2.1. Quantum-Based RF E-Field Sensing

**Figure 1:**
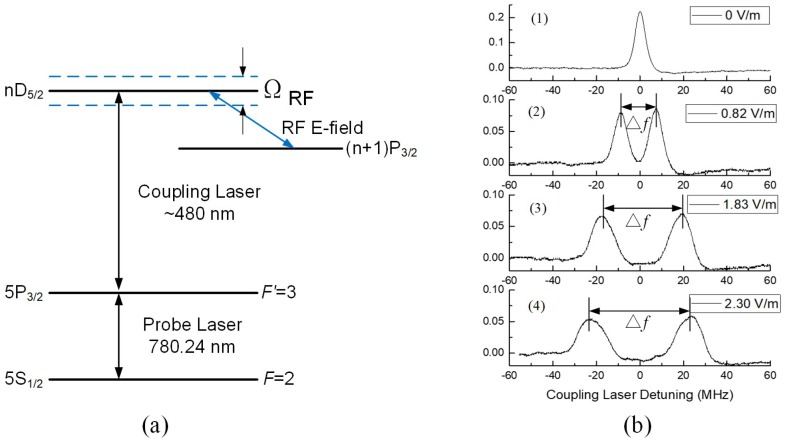
(**a**) Energy level of rubidium atoms involved in the radio-frequency (RF) electric field (E-field) measurement; (**b**) measured probe transmission spectroscopy at different E-field strengths as a function of the coupling laser frequency detuning.

The energy level of ^87^Rb atoms used in our measurements is illustrated in Figure 1a, where a ~780 nm probe laser is used for a D2 transition from ground state 5S_1/2_(*F* = 2) to intermediate state 5P_3/2_(*F*′ = 3), and a coupling laser of ~480 nm excites atoms from 5P_3/2_(*F*′ = 3) to a specific Rydberg state *n*D_5/2_, where *n* is the corresponding principle quantum number. As such, the Rydberg atoms can be prepared using this ladder-type configuration. By tuning the laser power and focusing the laser beams so they overlap, with a weak probe laser and a counter-propagating strong coupling laser, a phenomenon known as EIT [6] can be achieved, where the optical property of atomic gas changes from being absorbed to transparency. The first curve in Figure 1b shows a typical measured EIT signal as a function of the coupling laser frequency detuning (Δ*c*).

Atoms producing the EIT can be used as an active sensor for optically detecting the RF E-field. A specific E-field in the RF range can couple some high-lying Rydberg states, such as *n*D_5/2_ and (*n* + 1)P_3/2_ in Figure 1a, which results in quantum coherence in the probe transmission. From a phenomenon point of view, EIT spectroscopy is split by a RF E-field coupled Rabi frequency Ω_RF_ defined in Equation (1). This is known as Autler-Townes (AT) splitting [7].

(1) $$\Omega_{\mathrm{RF}}=\frac{\mu |E|}{\hbar },$$

where *|E|* is the E-field strength, ℏ is the known reduced Planck’s constant whose value will be frozen in the revision of SI [23], and *μ* is the transition matrix element that can be calculated using information of corresponding resonant Rydberg states.

**Figure 2:**
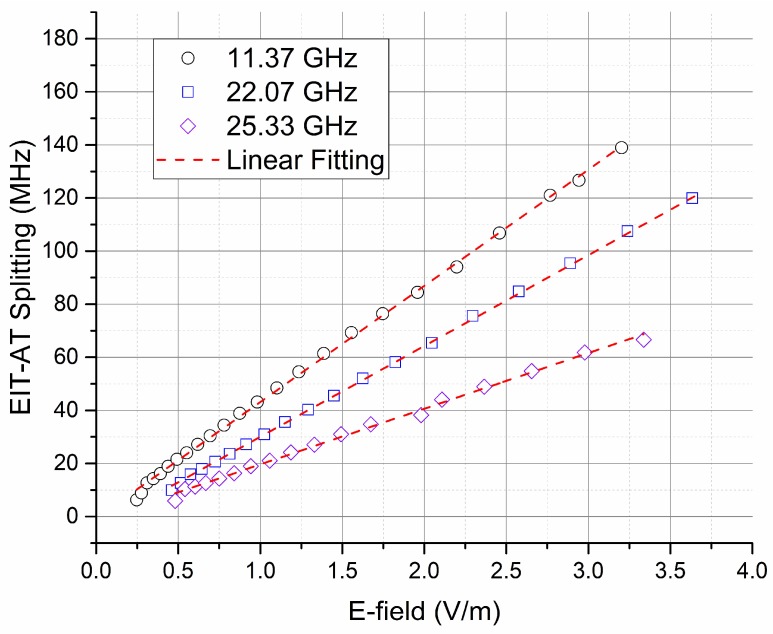
Measured Autler-Townes (AT) splitting with respect to different RF E-field illumination at 11.37 GHz, 22.07 GHz, and 25.33 GHz, where Rydberg transitions 57D_5/2_ → 58P_3/2_, 46D_5/2_ →47P_3/2_, and 44D_5/2_ → 45P_3/2_ of ^87^Rb atoms are used, respectively. A strong linear dependence of field strength, *|E|,* and the splitting frequency readout, Δ*f,* was achieved. EIT, electromagnetically-induced transparency.

Figure 1b presents typical AT splitting spectroscopy at different E-field illumination. A stronger E-field results in a larger splitting. The quantum mechanism allows measurement of E-field strength via the splitting readout (Δ*f*). Due to the Doppler mismatching between the counter-propagating probe and coupling lasers, the frequency splitting is scaled by a ratio *K* = *λ*_p_/ *λ*_c_ when scanning the probe laser while locking the coupling laser, where *λ*_p_ and *λ*_c_ are the wavelengths of the probe and the coupling lasers, respectively. On the contrary, scanning the coupling laser while locking the probe laser produces *K* = 1. The quantum relationship specified in Equation (2) bridges the E-field strength *|E|* and the optical frequency interval Δ*f*, which can be measured accurately using current techniques. The linearity dependence of field strength *|E|* and measured splitting readout Δ*f* has been validated in Figure 2, where Rydberg transitions 57D_5/2_ → 58P_3/2_, 46D_5/2_ → 47P_3/2_, and 44D_5/2_ → 45P_3/2_ were used for 11.37 GHz, 22.07 GHz, and 25.33 GHz E-field measurement, respectively.

(2) $$|E|=\frac{\hbar }{\mu }\Omega_{\mathrm{RF}}=2\pi K\frac{\hbar }{\mu }\Delta f.$$

By preparing atoms in different Rydberg states by tuning the coupling laser wavelength, the frequencies of resonant E-field coupling nearby Rydberg states can cover 1 GHz up to 500 GHz [10], with the corresponding principal quantum number of Rydberg states ranging from 20 to 100. Combining with other techniques, such as the RF detuning method [24], two-photon transition [25,26], and by using mixed atomic gas [27], this quantum mechanism allows ultra-broadband RF E-field measurement.

### 2.2. Experiment Apparatus and Configuration

**Figure 3:**
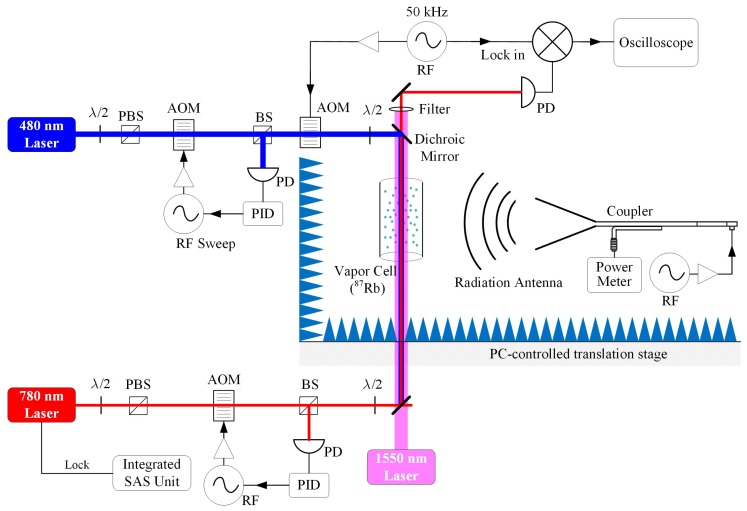
Schematic of experimental setup: AOM, acousto-optic modulator; BS, beam splitter; PBS, polarizing beam splitter; PD, photo-diode detector; PID, proportional–integral–derivative controller; SAS, saturated absorption spectrum; *λ*/2, half wave plate.

The measurement setup is shown in Figure 3. In a room-temperature atomic vapor cell, rubidium (^87^Rb) atoms were excited by the counter-propagating probe and coupling lasers via a two-photon transition in a ladder-type configuration. The probe laser was generated from an external-cavity diode laser (ECDL) (Toptica DL Pro), and was frequency-locked to |5S_1/2_, *F* = 2>→|5P_3/2_, *F*′ = 3> transition via an integrated saturated absorption spectroscopy (SAS) unit of ^87^Rb atoms, resulting in a linewidth of ~100 kHz. The coupling laser was produced by a high-power doubled-frequency diode laser system (Toptica TA-SHG), and its frequency was scanned across the resonant frequency of Rydberg transition |5P_3/2_, *F*′ = 3>→|*n*D_5/2_> using an acousto-optic modulator (AOM). The power of the two lasers was stabilized using two proportional-integral-derivative (PID) controllers, separately, and a power deviation of less than 1% was achieved. In the following experiments, the power of the probe laser and the coupling laser was stabilized to 10 μW and 35 mW, respectively. The probe and coupling lasers overlapped counter-propagating, and their beams were focused to a waist-diameter of 100 μm and 150 μm, respectively. The transmission spectroscopy of the probe laser was detected by a high-speed photodiode detector (PD) and recorded by a digital oscilloscope. To improve the signal-to-noise ratio, the coupling laser was amplitude-modulated by a 50 kHz sine wave using another AOM, and the resulting probe transmission signal was demodulated by a lock-in amplifier. In addition, with a thermal coating using a high temperature paint (Pyromark) and 1550 nm high power laser illumination, the surface temperature of the vapor cell can be heated up to about 60 °C [28].

Following the construction of the RF E-field standard [11], the E-field was generated by a radiation antenna, and normally standard gain horn antennas were used. The antenna was fed by a vector signal generator (E8257D, Keysight, Santa Rosa, CA, USA) and the net feeding power to the antenna port is monitored with a calibrated directional coupler and a commercial thermistor power sensor (NRP 110T, Rohde & Schwarz, Munich, Germany). The vapor cell was placed in the far-field range of the antenna, and at the same height as the antenna aperture center. The vapor cell and antenna were mounted and supported using some low electromagnetic reflection material on a PC-controlled translation stage, which we used for scanning the E-field distribution inside a vapor cell. Some absorbers were set up around the vapor cell in order to eliminate any reflection from the surroundings.

## 3. Results

### 3.1. E-Field Strength Disturbance

The existence of a dielectric vapor cell can change the magnitude and spatial distribution of the incident fields. Such influences would be significant if an incident wave’s frequency coincides with the resonant frequencies of a vapor cell. The eigenmode is an intrinsic property of a resonant cavity, which depends only on the structure and material of a dielectric vapor cell. In this work, eigenmode analysis was adopted to understand and optimize the electromagnetic response of a vapor cell. The tool is available from commercial software, the High Frequency Structure Simulator (HFSS), which is based on the finite-element method. Three cuboidal vapor cells with different dimensions were modeled, which were all made of Pyrex with a measured permittivity (*ε*) of 4.532 and loss tangent of 0.022. Table 2 provides the detailed dimensions and the calculated resonant frequency results. Note that as the vapor cell was symmetric along the three axes, up to three degenerated modes may have existed at one resonant frequency.

**Figure 4:**
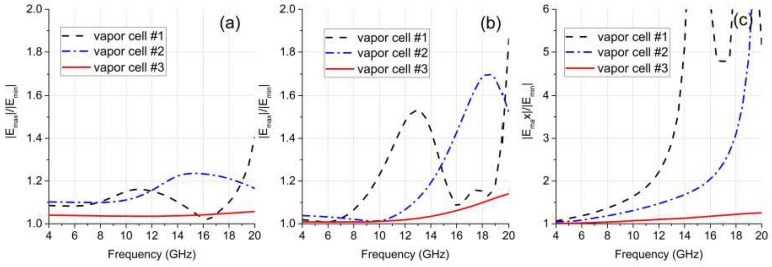
Computed field uniformity of the vapor cells from 4 GHz to 20 GHz, using the ratio of maximum to minimum field strength (|***E***_max_|/|***E***_min_|) over the calculation range as an indicator. The results of field distribution uniformity in the vapor cell center along the (**a**) *x*-axis, (**b**) *y*-axis, and (**c**) *z*-axis.

The incident plane wave propagated along the *z*-axis, whereas the polarization direction of the E-field was parallel to the *y*-axis. Figure 4 illustrates the uniformity of the E-field distribution in the centers of the vapor cells along three orthogonal directions (*x*-axis, *y*-axis, and *z*-axis). A ratio of maximum to minimum field strength (|*E*_max_|/|*E*_min_|) over the calculation range was used to characterize the field uniformity, where a value close to one means that the field is nearly uniformly distributed.

The field distribution results coincide with the eigenmode analysis normally up to the lowest resonant frequency, where the field distortion was relatively small. To minimize the cavity resonance effect, the incident wave frequency should be much lower than the resonant frequencies of the modes that can be excited. Their lower modes have different resonant frequencies. Vapor cells 1 and 2 had different outer sizes but the same wall thickness (1 mm). It is clear that a larger size resulted in lower resonant frequencies, meaning that it would be easier to disturb the field. This is consistent with the conclusion by Fan et al. [21]. Vapor cells 2 and 3 had the same inner size (8 mm) but different wall thicknesses. The lowest mode of vapor cell 2 was resonant at 18.01 GHz, whereas the lowest mode of vapor cell 3 was resonant at over 24.72 GHz. The reduction in wall thickness increased the lowest resonant frequency by 37.3% for the studied case, thus reducing the field disturbance.

**Figure 5:**
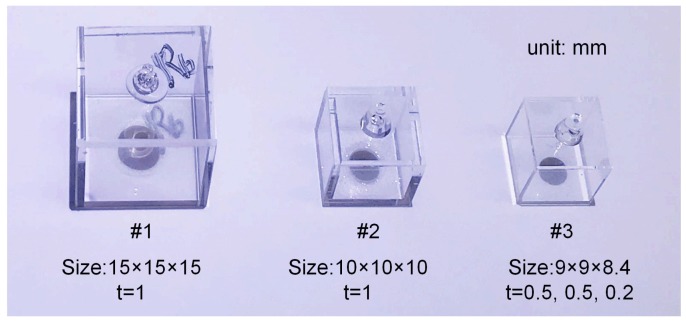
Photo of cuboidal vapor cells used in the experiments.

The vapor cells in Table 2 were fabricated using a Pyrex glass bonding technique. Figure 5 shows a photo of this. Sometimes it is difficult to fabricate a vapor cell with very thin walls, and so only two parallel walls perpendicular to the field propagation of vapor cell 3 were made as thin as 0.2 mm, whereas the others were 0.5 mm in thickness. The ^87^Rb vapor pressure was about 10^−5^ Pa.

**Figure 6:**
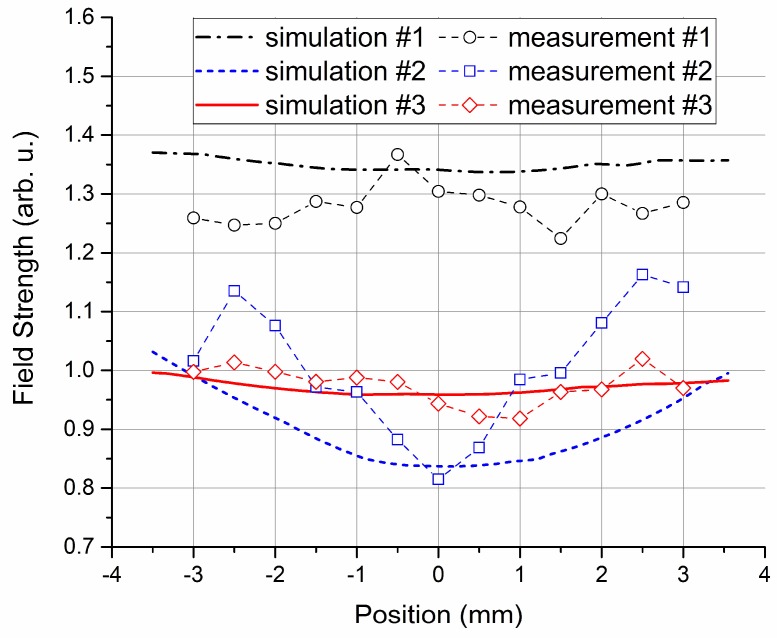
Internal field strength distribution in the center of different vapor cells along the *x*-axis with respect to different vapor cell geometries at 15.09 GHz. The results are compared in the overlapped range of these vapor cells, i.e., in a 7.0 mm segment.

The field strength distribution in the center of different vapor cells along the *x*-axis was measured using the setup illustrated in Figure 3. The applied 15.09 GHz E-field couples the Rydberg states 52D_5/2_ and 53P_3/2_. The results are given in Figure 6, together with the HFSS-based simulation results. It is obvious that the field strength distortion was reduced not only by reducing the size of the vapor cell, but also by reducing the wall thickness. The measurement results were consistent with the simulation prediction.

### 3.2. E-Field Polarization Disturbance

**Figure 7:**
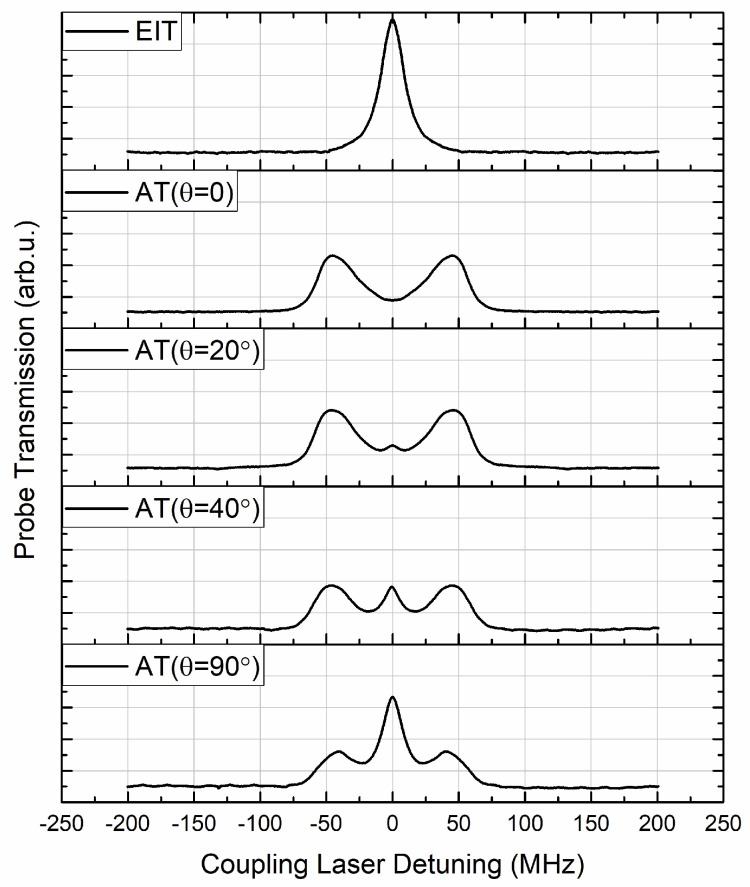
Typical measured transmission spectroscopy of the probe laser in conditions of different E-field polarizations. The top curve noted as “EIT” is the EIT spectroscopy of the probe laser without any incident RF E-field. The other curves are the AT splitting in the presence of a 15.09 GHz E-field. The lineshape differences are due to the relative angle (*θ*) between the E-field vector and laser polarization.

In the phenomenon of RF E-field-induced AT splitting within an EIT, the probe transmission spectroscopy is dependent on the laser and RF E-field polarization. Sedlacek interpreted this by considering all 52-level degenerate magnetic sublevels of each state, taking 5S_1/2_ → 5P_3/2_ → 53D_5/2_ → 54P_3/2_ as an example [9]. Typical EIT and AT splitting spectroscopy with and without the presence of RF E-fields are illustrated in Figure 7, where the first curve (noted as “EIT”) is the EIT spectroscopy of the probe laser without any incident RF E-field, and the other curves are the AT splitting in the presence of a 15.09 GHz E-field. The lineshape differences are due to variation in the relative angle (*θ*) between the E-field vector and laser polarization. In an ensemble of atoms, some will exhibit three-level behavior, and others will have four-level behavior, so experimental lineshape can be characterized as combinations of three-level EIT and four-level EIT-AT. The probe transmission at zero coupling detuning (Δ*c* = 0) increased as the relative polarization angle (*θ*) increased from 0° to 90°, and reached a maximum when *θ* was 90° (cross-polarized).

In most circumstances during E-field strength measurements, the probe and coupling lasers are co-linearly polarization aligned, and with the same polarization as the RF E-field. When scanning the field distribution inside a vapor cell, similar results to AT splitting spectroscopy, as in Figure 7, were obtained where the probe transmission at zero coupling detuning (Δ*c* = 0) varied at different positions inside a vapor cell. This indicates distortion of the RF E-field polarization.

An approximated equation of the probe transmission at zero coupling detuning (Δ*c* = 0), as a function of the relative angle of E-field vector and co-polarized laser polarization, is given in [9], which is represented here as Equation (3):

(3) $$T=1-\cos^{2}(\theta )\sin^{2}(\psi ),$$

where *θ* is the relative angle between the E-field vector and laser polarization and *ψ* is the angle between the E-field vector and the laser path. The probe transmission was normalized such that the maximum transmission was one.

**Figure 8:**
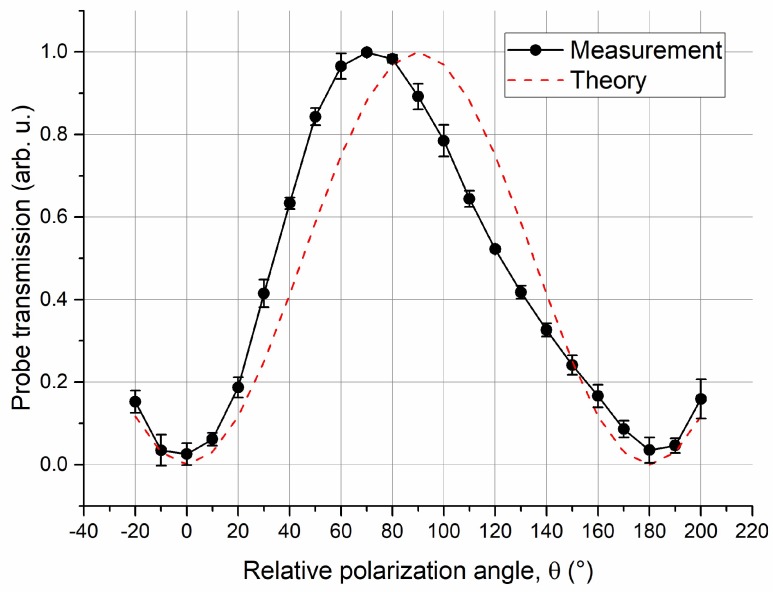
Probe transmission at zero coupling detuning (Δ*c* = 0) for different angles between the laser polarization and the RF E-field vector, where the solid line is the averaged result of multiple measurements and the related experimental standard deviation, and the dashed line represents the theoretical results.

The dependence of the probe transmission at Δ*c* = 0 on the laser polarization and E-field vector was validated by measurement at 15.09 GHz. By rotating either the probe or coupling laser polarizations, it was possible to align the laser polarization by checking the EIT signal level. The polarization subsequently remained parallel. The vapor cell was positioned in the far-field range of a standard gain horn antenna, whose polarization was clearly defined, and the polarization of the E-field was perpendicular to the laser path, which caused *ψ* to be 90°. By turning the laser polarization, the probe transmission at zero coupling detuning (Δ*c* = 0) was as low as a signal floor of probe transmission, and this means that the laser and E-field were polarization matched (*θ* = 0). Using this as a reference, the probe transmission at Δ*c* = 0 as a function of relative polarization angle *θ* was measured. Figure 8 shows the results of multiple measurements, which is consistent with the theoretical relationship determined by Equation (3).

**Figure 9:**
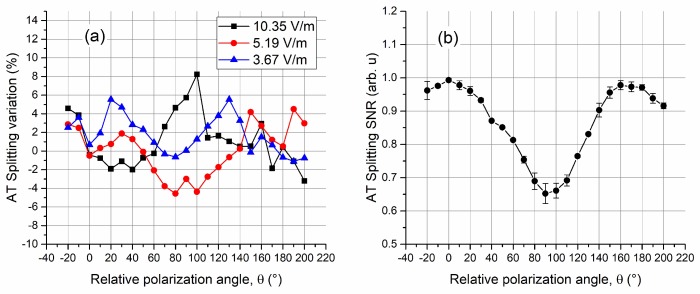
Influence of relative angles between laser polarization and RF E-field vector on the E-field strength measurement: (**a**) the variation in AT splitting determination relative to the splitting when laser polarization and RF E-field vector are well matched, and (**b**) normalized signal-to-noise ratio of the AT splitting.

The variation in probe transmission due to the polarization mismatch of the E-field and lasers can influence the determination of the field strength. Fundamentally, the polarization mismatch is a superposition of three- and four-level behaviors [9], which results in an increase in the probe transmission at zero coupling detuning (Δ*c* = 0), and a consequent decrease in signal strength of AT splitting on both sides, as Figure 7 indicates. The variation in the lineshape of the probe transmission spectroscopy may cause an error in the splitting measurement. Figure 9a shows the variation in AT splitting determination at 15.09 GHz with respect to the relative angle *θ*. The results are presented in a relative variation percentage compared with the AT splitting determined when the E-field and lasers were polarization-matched (*θ* = 0). Results for different RF E-field strength measurements indicate that the maximum variation normally occurs at the cross polarization (*θ* = 90°). When rotating the probe and coupling laser polarizations around their propagation axes from *θ* = −20° to *θ* = 200°, the maximum variation of 8.2% occurred for 10.35 V/m measurement, and the standard deviations of all the values were 2.8%, 2.6%, and 2.1% for 10.35 V/m, 5.19 V/m, and 3.67 V/m measurements, respectively.

The signal-to-noise ratio (SNR) of the AT splitting decreased with an increase in relative polarization angle *θ*. Figure 9b provides the measured SNRs with respect to different *θ* values, together with the experimental standard deviations of multiple measurements. The results were normalized to a maximum signal level when the E-field and optical field were polarization-matched (*θ* = 0). The trend was nearly opposite with respect to the variation of the probe transmission at zero coupling detuning (Δ*c* = 0). This can be interpreted by the superposition of an EIT and an EIT-AT spectroscopy from a phenomenon point of view. The SNR decreased by about 35% when the relative polarization angle (*θ*) tuned from 0° to 90°. This may become a serious issue for very weak E-field measurement because the increase in probe transmission at zero coupling detuning (Δ*c* = 0) complicates the determination of a small splitting.

**Figure 10:**
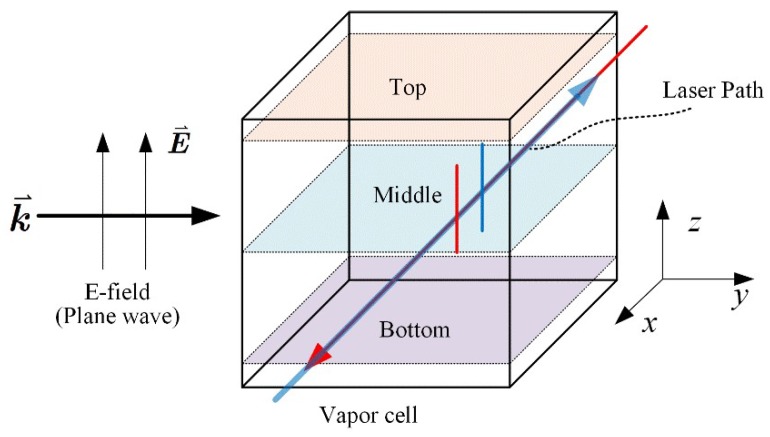
The simulation model for investigating the polarization distortion inside a cuboidal vapor cell, where the propagation ($\vec{k}$) and polarization ($\vec{E}$ ) of the RF E-field and lasers are illustrated with a systematic coordinate. The calculation focused on the three typical layers located at the top, middle, and bottom of the vapor cell.

**Figure 11:**
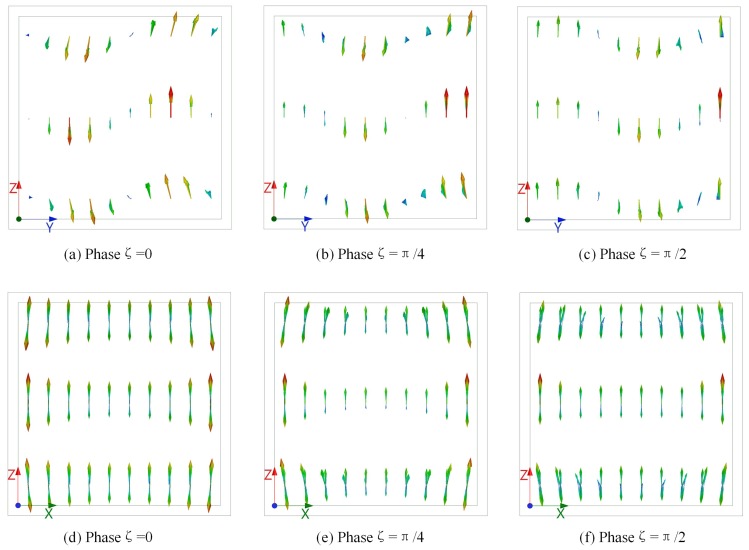
Simulation results of RF E-field vector distribution inside a 20 mm cuboidal vapor cell at 15 GHz. Vector distribution projecting on the *yz*-plane while observing along the *x*-axis, with relative phases of incident E-field of (**a**) 0, (**b**) π/4, and (**c**) π/2, respectively. Vector distribution projecting on the *xz*-plane while observing along the *y*-axis, with relative phases of (**d**) 0, (**e**) π/4, and (**f**) π/2.

The finite-element numerical simulation was performed to further study the polarization distortion effect inside a vapor cell. Figure 10 shows a numerical model of a 20 mm cuboidal vapor cell with 1 mm wall thickness, made of Pyrex glass. An ideal vacuum boundary condition was set both inside and outside the vapor cell. The incident plane wave E-field vector runs parallel with the *z*-axis propagated along the *y*-axis. The probe and coupling lasers counter-propagated along the *x*-axis with a parallel polarization to the incident E-field. The E-field vector distributions at 15.09 GHz in three different layers, as shown in Figure 10, were calculated. The top, middle, and bottom layers were located in planes *z* = 18 mm, *z* = 10 mm, and *z* = 2 mm, respectively. Figure 11 illustrates the E-field vector distribution observed along the *x*-axis and *y*-axis, at different phases of the incident field. It was obvious that the polarization of the E-field was distorted inside a vapor cell, and it was no longer uniformly distributed along the E-field and laser propagation path. The vector projection onto the *yz*-plane and *xz*-plane indicated that the polarization remained much better in the middle layer than the top and bottom layers. This conclusion was validated with vapor cell models of various geometries.

Following the field strength distribution measurement, by moving the vapor cell and the radiation antenna simultaneously with a PC-controlled translation stage (which is equivalent to scanning the lasers relatively), the probe transmission spectroscopy at sampling positions in the middle and bottom layers were measured. The variation in probe transmission at the zero coupling detuning (Δ*c* = 0) was measured to determine the polarization distortion of the RF E-field inside the vapor cell. As a nonlinear relationship specified in Equation (3) and demonstrated in Figure 8, and due to the low SNR of the probe transmission at Δ*c* = 0, it was not easy to accurately detect the variation in the probe transmission near polarization-matching (*θ* = 0). The measurement started with a polarization offset of *θ*_0_ = 45°, and then the relative variations with respect to *θ*_0_ were determined.

There was little variation in the internal E-field polarization in laser propagation of the middle layer, as shown in the simulation results in Figure 11. The angle *ψ* nearly remained constant at 90°, and thus Equation (3) can be simplified as Equation (4). For the cases where *ψ* varies from 90°, as the vector distribution in the top and bottom layers, *θ* determined by Equation (4) will be underestimated.

(4) $$T=1-\cos^{2}(\theta ).$$

**Figure 12:**
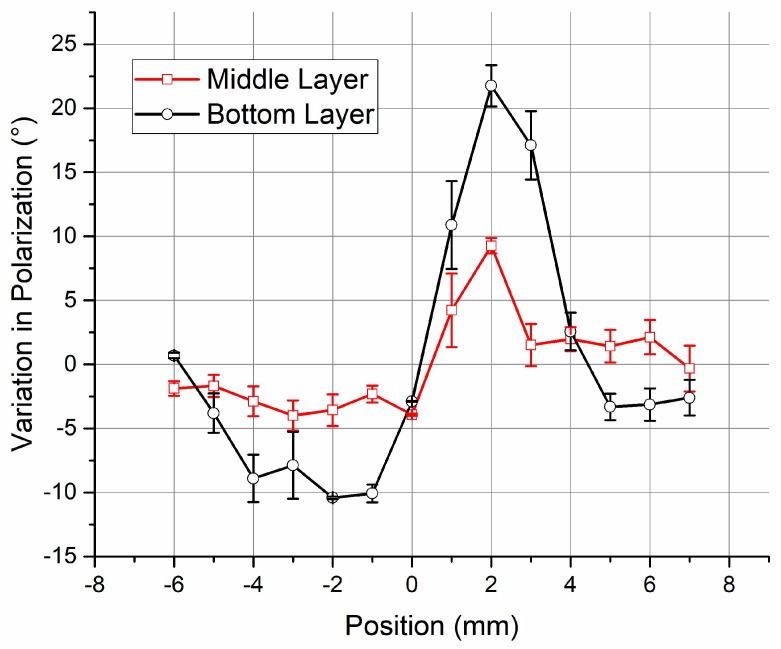
The variation in relative angles between the laser polarization and the RF E-field vector when the laser was passing through different positions of the vapor cell. The red curve (noted as “Middle Layer”) and the blue curve (noted as “Bottom Layer”) are the results of scanning the middle and the bottom of the vapor cell, respectively.

The probe transmission at the zero coupling detuning (Δ*c* = 0) was normalized such that when the maximum (when *θ* equals 90°) was one, the variations in internal E-field polarization were determined by Equation (4). Figure 12 shows the relative polarization variation in the middle and bottom layers as a function of sampling positions. Even the result in the bottom layer was underestimated; the variation was much bigger than that in the middle layer. Multiple measurements have validated our simulation. For the bottom layer, the experimental standard deviation and the maximum results of *θ* were 9.3° and 21.8°, respectively. According to the results in Figure 9a, this may cause a 1–5% error in field strength determination. Using atoms in the middle of a vapor cell may reduce the polarization distortion and improve the measurement accuracy.

## 4. Conclusions

Due to the cavity RF resonance and electromagnetic scattering effect, the presence of a dielectric-vapor cell varies the magnitude, polarization, and spatial distribution of the incident E-field in a vapor cell. The investigation and optimization of a vapor cell in terms of E-field distortion are necessary for developing a self-calibrated quantum E-field sensor. Both the field strength distortion and depolarization of rubidium vapor cells were characterized by measuring the E-field vector distribution inside different vapor cells, and a full-wave numerical simulation and an eigenmode analysis were used to determine optimization solutions. The field strength distortion effect was reduced by diminishing the electrical size (*D*/*λ*) of a vapor cell, and a reduction in wall thickness further minimized such field disturbance under the same *D*/*λ* ratio. The polarization of the incident E-field was determined by measuring the probe transmission at zero coupling detuning. In a word, to minimize field distortion, the vapor cell should be as small as possible (compared with RF wavelength), as thin as possible, made of a low-permittivity material, and the middle of the sensor in the direction of the incident E-field vector is the suitable position for field sensing. This work promotes the atom-based RF E-field measurement and may be useful in other related quantum RF sensing as well. Efforts will be made in the implementation of new design techniques, such as the geometry optimization, the frequency selective surface (FSS), and metamaterials, to further minimize the RF field distortion.

## Figures and Tables

**Table 1:** Comparison of radio-frequency (RF) electric field (E-field) probes.

	Conventional Probe [11] (Dipole Antenna-Based)	Quantum Sensor
**Frequency range**	<100 GHz	~1–500 GHz (single sensor) [10]
**Sensitivity**	~10^−1^ V/m	~10^−10^ V/m (quantum limit) [12,13]
**Uncertainty**	5–10% (0.5–1 dB)	0.5% (potentially) [8]
**Spatial resolution**	~*λ*/2	~*λ*/100 [14,15,16]
**Physical dimension**	Relatively large, frequency-dependent	Chip-scale, micro vapor cells [17,18,19]
**Calibration**	Needs calibration in a standard RF E-field	Self-calibrated [10]
**Traceability**	Complex	Clear, linking to Planck’s constant

**Table 2:** Vapor cells with different sizes and their resonant frequencies obtained using eigenmode analysis.

Number	Dimensions (mm)	Resonant Frequencies (GHz)
1	15 × 15 × 15, *t* ^1^ = 1	13.02, 14.87, 19.17, 20.01, 20.04
2	10 × 10 × 10, *t* = 1	18.01, 19.77, 24.68, 25.65, 26.44
3	8.4 × 8.4 × 8.4, *t* = 0.2	24.72, 29.67, 38.88, 39.03, 41.79

^1^*t* is wall thickness, and vapor cell sizes are described using outer dimensions.
